# Assessing the Effect of Training on the Cognition and Brain of Older Adults: Protocol for a Three-Arm Randomized Double-Blind Controlled Trial (ACTOP)

**DOI:** 10.2196/20430

**Published:** 2020-11-24

**Authors:** Arnaud Boujut, Samira Mellah, Maxime Lussier, Samantha Maltezos, Lynn Valeyry Verty, Louis Bherer, Sylvie Belleville

**Affiliations:** 1 Research Center, Institut universitaire de Gériatrie de Montréal Montréal, QC Canada; 2 Department of Psychology Université de Montréal Montréal, QC Canada; 3 Department of Medicine Université de Montréal Montréal, QC Canada; 4 Research Center, Institut de cardiologie de Montréal Montréal, QC Canada; 5 Department of Neuroscience Université de Montréal Montréal, QC Canada

**Keywords:** cognitive training, working memory, brain plasticity, aging, cognitive reserve

## Abstract

**Background:**

To prevent age-related cognitive impairment, many intervention programs offer exercises targeting different central cognitive processes. However, the effects of different process-based training programs are rarely compared within equivalent experimental designs.

**Objective:**

Using a randomized double-blind controlled trial, this project aims to examine and compare the impact of 2 process-based interventions, inhibition and updating, on the cognition and brain of older adults.

**Methods:**

A total of 90 healthy older adults were randomly assigned to 1 of 3 training conditions: (1) inhibition (Stroop-like exercises), (2) updating (N-back-type exercises), and (3) control active (quiz game exercise). Training was provided in 12 half-hour sessions over 4 weeks. First, the performance gain observed will be measured on the trained tasks. We will then determine the extent of transfer of gain on (1) untrained tasks that rely on the same cognitive process, (2) complex working memory (WM) measurements hypothesized to involve 1 of the 2 trained processes, and (3) virtual reality tasks that were designed to mimic real-life situations that require WM. We will assess whether training increases cortical volume given that the volume of the cortex is determined by cortical area and thickness in regions known to be involved in WM or changes task-related brain activation patterns measured with functional magnetic resonance imaging. Dose effects will be examined by measuring outcomes at different time points during training. We will also determine whether individual characteristics moderate the effect of training on cognitive and cerebral outcomes. Finally, we will evaluate whether training reduces the age-related deficit on transfer and brain outcomes, by comparing study participants to a group of 30 younger adults.

**Results:**

The project was funded in January 2017; enrollment began in October 2017 and data collection was completed in April 2019. Data analysis has begun in June 2020 and the first results should be published by the end of 2020 or early 2021.

**Conclusions:**

The results of this study will help understand the relative efficacy of 2 attentional control interventions on the cognition and the brain of older adults, as well as the moderating role of individual characteristics on training efficiency and transfer.

**Trial Registration:**

ClinicalTrials.gov NCT03532113; https://clinicaltrials.gov/ct2/show/NCT03532113

**International Registered Report Identifier (IRRID):**

DERR1-10.2196/20430

## Introduction

### Background

Slowing age-related cognitive decline is a central concern for the prevention of pathological aging and loss of autonomy. Cognitive training has been identified as having significant potential in this context [1]. Many studies have identified working memory (WM) as a target for cognitive training because it is considered a foundational element of cognition. WM maintains and manipulates online information and supports many complex cognitive activities including language comprehension, reasoning, and mathematical abilities. Furthermore, aging and neurodegenerative diseases can impair WM, which has a negative impact on the ability to carry out high-level cognitive tasks. WM is a multicomponent system and relies on different attentional control processes (see [2] for a review), some of which have been the target of WM training programs. The goal of this study is to compare the effect of 2 attentional control training programs in older adults, each targeting major WM processes: inhibition and updating. A side-by-side comparison of inhibition and updating training programs will determine their respective and comparative impact.

We propose to measure the training program’s effect on proximal measures, as well as on transfer tasks and brain outcomes. Moving from proximal to transfer tasks is critical. A large number of studies have used updating training in older adults and found improvements on proximal tasks, (eg, [3-5]), with some generalization to switching tasks (eg, [6]). A few studies reported that inhibition training was effective when measured with inhibition tasks that were similar to those done in training [7,8]. However, little evidence was found to support generalization to untrained tasks. Thus, inhibition and updating training improve tasks that are similar to the training, but little is known about their effect on more complex tasks or comparative efficacy. It is important to determine whether improving one attentional process improves another, which would suggest within-WM generalization. In addition, tasks typically used to measure transfer rely on laboratory-based cognitive tasks, designed to reflect fine cognitive functions or processes. They are therefore not representative of the complexity present in everyday life. Thus, in addition to traditional complex WM tasks, transfer will be assessed using virtual reality, which can be used to reproduce real-life situations [9,10].

Neuroimaging can also provide critical information regarding brain processes engaged by the 2 training programs. Updating has been consistently associated with activation of the frontal and parietal lobes, including the dorsolateral and ventrolateral prefrontal cortices, the inferior parietal lobules, insula, and the premotor and supplementary motor areas [11-13]. Inhibition processes have been associated with the anterior cingulate cortex, middle frontal gyrus, inferior frontal regions, and parietal areas [14-16]. Functional neuroimaging studies have shown that updating training increases activation in the striatum, while reducing activation in areas of the frontal and parietal lobe, as well as in the anterior cingulate and temporal cortices, which would reflect better neural efficiency following training [17-19]. Less is known about inhibition training, but it has been found to be associated with increased cortical thickness [7] and decreased activation in the inferior frontal gyrus [7,20]. Given that updating and inhibition are both part of the same WM system, it is important that we compare the effects of different process-based training programs within a single experimental design. Neuroimaging will also be used to assess whether differences in brain structure or function at baseline explain interindividual differences in training efficacy as proposed in prior studies [18,21,22].

In addition, effects of cognitive interventions may vary because of individual factors influencing the rate and magnitude of training gain [23-27]. Pathophysiological factors, such as the volume of white matter lesions, are known to impair brain plasticity and reserve in older adults [28] and may modulate the efficacy of cognitive interventions. Efficacy may also be moderated by sex [29], intracranial volume [30], and genetics, as these factors affect resilience to cognitive aging. For example, a common single-nucleotide polymorphism (Val^66^Met) from the brain-derived neurotrophic factor (BDNF) tends to decrease BDNF and reduce neuroplasticity. However, very little is known about the influence of BDNF polymorphism on the efficacy of WM training for cognition and brain health. WM training could be more beneficial for the Met carriers because they show a reduced baseline performance for attentional control performance compared to the Val homozygotes. However, only the Val homozygotes show significant reduction in performance over a 10-year span [31], suggesting that they may also respond well to WM training. Catechol-O-methyltransferase (COMT) Val^158^Met is involved in dopamine degradation, which contributes to frontal modulation and cognition. For instance, carriers of the Val allele of the COMT polymorphism demonstrate reduced baseline performance [32,33], but may show larger performance gains from WM training than carriers of the Met allele [34]. Meanwhile, it was found that the brain activation decreases more in the prefrontal cortex of Met carriers after WM training [35]. Moreover, apolipoprotein ε4 (APOE-ε4) is known to increase the risk of Alzheimer disease.

### Objectives

The general goal of the study is to compare 2 process-based interventions, inhibition and updating WM training, and assess their effect on the cognition and brain of cognitively healthy older adults relative to an active control condition. There are 6 objectives to the study: (1) measure the gains on the trained tasks; (2) determine transfer of gain on (i) proximal measures that are not trained directly but reflect the trained process, (ii) complex WM measures, and (iii) virtual reality tasks designed to reflect WM in real life; (3) identify the effect of WM training on cortical thickness and task-related brain activation in regions known to be involved in updating or inhibition or both; (4) assess whether cognitive (eg, pretraining cognition), psychosocial (lifestyle, education, motivation), and biological markers (eg, white matter lesions, sex, genotype) moderate the effect of training on cognitive and brain outcomes; (5) examine dose effects by measuring training, transfer, and cerebral outcomes at different time points; and (6) assess if training reduces the age-related deficit on cognitive and cerebral measures.

### Hypotheses

It is hypothesized that older participants enrolled in WM training will show larger cognitive gains than those in the active control condition, and that gains will be specific to the cognitive process trained. We expect brain changes to be observed in regions that are associated with the trained process, with an overall decrease in activation due to improved neural efficiency for both functional magnetic resonance imaging (fMRI) tasks. It is expected that both trainings improve brain and performance parameters in older adults, which will increase to the levels of younger adults. Finally, it should be possible to identify individual factors that moderate the magnitude of the effect. The intervention may especially benefit participants with less advantageous cognitive and genetic profiles. However, these participants may require additional training for the intervention to be effective [26].

## Methods

### Trial Registration and Reporting Guidelines

The Attentional Control Training in Older People (ACTOP) study is registered with the US National Institutes of Health clinical trials registry (ClinicalTrials.gov identifier NCT03532113). The methodology of the protocol follows the Standard Protocol Items: Recommendations for Interventional Trials (SPIRIT) guidelines [36]. All the collected data are stored using an anonymized protocol.

### Study Design

The study design presented in Table 1 is a randomized double-blind controlled trial where older participants are randomly assigned to 1 of 3 parallel groups: inhibition, updating, and active control. In line with prior empirical work from our team showing transfer effects following attentional control training in older adults [37], training was provided in 12 30-minute training sessions over 4 weeks. All transfer measurements were completed once by a group of younger adults who did not receive the training. This is used to assess whether training helps increase performance and brain status of older adults to the level of younger adults. Given the large number of appointments, participants were recruited and trained in 5-6 waves. The study was carried out at the Research Center of the Institut universitaire de gériatrie de Montréal (CRIUGM).

**Table 1:** Study design.

Schedule of events			Study period											
			Screening, baseline, and pretest		Intervention	Posttest 1	Intervention	Posttest 2		Intervention	Posttest 3	Intervention	Posttest 4	
Timepoint			V1	V2	V3–V5	V6	V6–V8	V9	V10	V11–V13	V14	V14–V16	V17	V18
**Enrollment**														
	Eligibility screen		X											
	Informed consent		X											
	Confirmation of eligibility			X										
	Randomization			X										
**Interventions**														
	Updating				X		X			X		X		
	Inhibition				X		X			X		X		
	General knowledge				X		X			X		X		
**Assessments**														
	**Clinical Assessments**													
		Montreal Cognitive Assessment (MoCA)	X											
		Logical Memory Test	X											
		Geriatric Depression Scale (GDS)	X											
		Ischemic Index	X											
		Cognitive Reserve Proxy Questionnaire (CRQ)	X											
**Training outcomes**														
	**Inverse efficiency score**													
		Updating			X		X			X		X		
		Inhibition			X		X			X		X		
**Proximal outcomes**														
	**Updating composite measure**													
		Keep track	X					X					X	
		Running span	X					X					X	
	**Inhibition composite measure**													
		Stroop Victoria	X					X					X	
		Anti-saccade	X					X					X	
**Complex working memory outcome**														
	**Working memory transfer**													
		Alpha span	X			X		X			X		X	
		Reading span	X			X		X			X		X	
	**Virtual car ride task composite measure**													
		Verbal memory	X			X		X			X		X	
		Visual detection	X			X		X			X		X	
**Brain structure**														
	Regional gray matter volume			X					X					X
	Cortical thickness			X					X					X
	Intracranial volume			X					X					X
	White matter lesions			X					X					X
**Brain activations**														
	Updating related			X					X					X
	Inhibition related			X					X					X
Salivary sample									X					

### Study Population and Eligibility Criteria

A total of 90 community-dwelling older adults (age 60-85) and 30 younger adults (age 20-35) were enrolled in the study. Older adults and younger adults were recruited in the Montreal area through advertisements in community centers, associations, local newspapers, and CRIUGM’s participants registry (*Banque de participants du* *CRIUGM*). In addition, students, who were enrolled in an undergraduate laboratory course and wished to help with the study recruited younger adults among their acquaintances. Younger and older adults with similar educational levels were recruited in order to reduce the cohort effect on education.

Participants with the following criteria were included in the study: right-handed, fluent in French, and sufficient visual and auditory acuity to undergo neuropsychological testing. Participants were only included if they performed above the cut-offs on the delayed recall portion of the Logical Memory Test of the Wechsler Memory Scale for older adults. Performance on the Logical Memory Subtest was considered normal based on the following education-adjusted cut-off scores used in the Alzheimer’s Disease Neuroimaging Initiative (ADNI) study: 9 or more for 16+ years of education; 5 or more for 8-15 years of education; 3 or more for 0-7 years of education [38-40].

Participants were excluded from the study if they had received a diagnosis of a disease or injury of the central nervous system (ie, moderate to severe chronic static leukoencephalopathy [including previous traumatic injury], multiple sclerosis, neurodevelopmental disorders, subdural hematoma [past or current], subarachnoid hemorrhage, primary cerebral tumor or cerebral metastases, epilepsy, dementia or other neurodegenerative diseases, stroke, intracranial surgery or major surgery within the last 2 months), alcoholism or substance abuse, general anesthesia in the past 6 months, serious comorbid conditions, major depression or anxiety, schizophrenia, or other major psychiatric disorders (eg, bipolar disorder). Participants were also excluded if they were unable to undergo an MRI scan, due to medical contraindications, or tolerate the procedure. Older adult participants were excluded if they reported subjective cognitive decline [41], such as feeling their memory is worse than it used to be and that it worries them, or if they had previously participated in structured attentional control training. Despite the time required for the participants to carry out the study, no change in the habits of daily living was required, except to abstain from cognitive training during the intervention. The younger adults were only included in the study if they were no longer full-time students. This constraint was intended to increase the diversity of age and education in our sample of younger participants in order to better match the diversity of our sample of older subjects. Moreover, it was not possible to determine the final level of education of the students, many of whom were actively involved in training programs that have made them experts in metacognitive strategies.

### Procedure

After a first telephone contact to assess global eligibility, potential participants were invited to the laboratory to consent to participate in the study, complete standardized clinical and neuropsychological assessments, and the outcome measurements for the baseline (V1; see list in Table 1). A brain imaging examination for the baseline was completed the following week (V2). Eligible participants were given CAD 50 (~US $38) at each brain imaging session, for a total of CAD 150 (~US $114) for older adults and CAD 50 (~US $38) for younger adults.

Data on proximal cognitive and brain outcomes were collected at 3 timepoints: no more than 2 weeks prior to training for the baseline (PRE), between training sessions 6 and 7 (POST2), and no more than 1 week following the end of training (POST4) for the postbaseline tests. Complex WM outcomes were measured at 5 time points: (1) at PRE; (2) after the third training session (POST1), (3) at POST2, (4) after the ninth training session (POST3), and at (5) POST4. The complex WM outcomes at POST1 and POST3 were completed on the same day as a training session to reduce travel and the number of visits to the laboratory. The standard duration of the study was 8 weeks and involved 18 appointments. Participants were informed that the study must be performed without discontinuity. A postponement of up to 1 week was exceptionally authorized in the event of unforeseen circumstances. To limit the influence of the circadian rhythm on performance, training sessions and assessments were performed at similar times of day (ie, morning or afternoon) for a given participant. In addition, at the end of the first appointment, a personalized calendar containing all dates and durations of appointments was given to each participant in paper format. A reminder email (or a phone call made if no email address was available) was also sent the day before each MRI appointment.

### Randomization and Blinding

Participants meeting eligibility criteria were randomly assigned to 1 of the 3 interventions. An independent project coordinator, who was not involved with the enrollment process, cognitive assessments, or interventions, generated blind randomized samples without replacement. Randomization allocated eligible participants individually to 1 of the 3 training conditions using a computer-based random digits program (ie, one participant at a time as they enter the study). In this double-blind study design, participants were blind and unaware of the experimental and control conditions, as they were instructed that different capacities were trained in different programs. Assessors were blind to the participants’ intervention condition. If a participant accidentally shared information that may identify their intervention group to an assessor, the incident was recorded and a different assessor was assigned for this participant for the following assessments. Training supervisors were aware of the intervention assigned to the participants. All participants were assigned 2 anonymized identification numbers: one known by the training supervisors only and the other known by the assessors only. For simplicity, the counterbalancing for the tasks’ versions of transfer outcomes was done upstream for each age group separately, using a replicated Latin square design, regardless of the older adults’ intervention.

### Interventions

The 2 experimental training conditions (inhibition and updating) were provided by the Neuropeak web platform (Lussier M et al, unpublished data) using a Samsung Galaxy Tab 2 (Android version 4.2.2).

**Figure 1:**
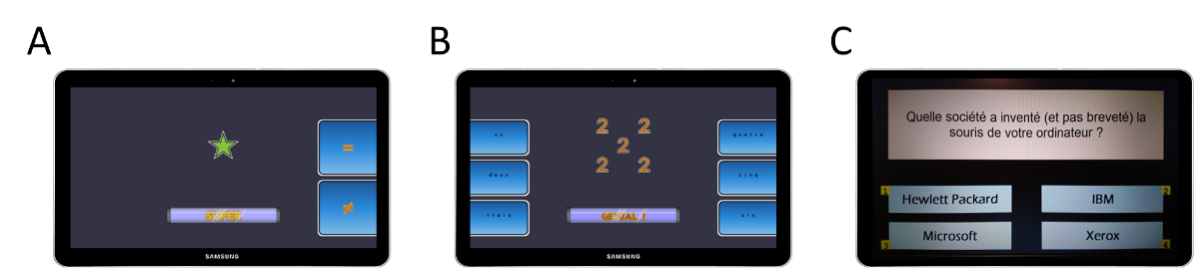
Illustration of the training exercises used to train attentional control: (A) in this Neuropeak updating exercise, the task consisted of indicating whether the current symbol (eg, a star) matches (or does not match) the previously displayed symbol in 1-, 2-, or 3-back position. (B) In this Neuropeak inhibition exercise (incongruent trials), the task consisted of indicating the number of copies (eg, 5) of the digit displayed in the center of the screen (eg, 2). (C) In this general knowledge quiz game, the task consisted of indicating the number of the correct answer (eg, 4) to the question displayed in the upper part of the screen.

The updating training involved 2 N-back-type exercises (1-2- and 3-back) with different sets of stimuli. Both sets were performed during each of the 12 training sessions. The first set comprised stimuli made from digits (1 to 9) and the second comprised symbols (moon, planet, star, dog, bird, snake). The stimuli were displayed on the center of the screen, one by one, at the rate of 4 seconds per item (Figure 1A). In both sets, participants were asked to indicate whether each item matches the one presented in the *n* position previously (eg, 3-6-3-9-9 wherein the second “3” is the only match in a 2-back block). Each round comprised 8 blocks grouped by N-back level and performed in the following order: 1-back (2 × 11 trials), 2-back (3 × 12 trials), and 3-back (3 × 13 trials). Each of the 8 blocks were set to include 40% “match” responses. Nonetheless, participants were able to reach the 3-back level only if their accuracy was equal or above 75% at the 2-back level. If they were below this percentage, they finished the round with a 1-back block instead. Participants were instructed to respond as fast as possible. “Match” and “Mismatch” buttons were permanently displayed on the right side of the screen and participants were required to answer with their right thumb.

Inhibition training also involved 2 Stroop-like exercises with different sets of compound stimuli. Both sets were performed during each of the 12 training sessions. The first set comprised compound stimuli made from digits (1 to 6) and the second included compound stimuli made from letters (D, F, H, L, S, T). The 2 different sets were used to reduce the stimulus–response dependency of the improvement in performance on tasks, and hence facilitate transfer. Stimuli was displayed in the center of the screen (Figure 1B). In the first set, participants were asked to count the number of items in each trial while in the second set, they were asked to identify the largest letter. In each set, they were presented 3 types of stimuli: congruent (eg, 5 copies of the digit “5” or a large “H” formed from smaller Hs), neutral (eg, five copies of the symbol “*” or a large “H” formed from smaller “*”), and incongruent (eg, 5 copies of the digit “3” or a large “L” formed from smaller Hs). Each of the 2 sets comprised 7 blocks. The first block contained 20 congruent compound stimuli, the second block contained 60 neutral stimuli, and the third 60 incongruent compound stimuli. Incongruent items require inhibiting the smaller stimuli to determine the larger one. The congruent and incongruent blocks were then repeated twice in alternance. Participants were instructed to respond as fast as possible while maintaining high accuracy. Response keys were displayed on both sides of the touchscreen, and participants responded with their thumbs. Participants were instructed that there was no time limit but responses taking longer than 4 seconds were not recorded to avoid the impact of outliers. A response immediately triggered the following target in order to reduce the contribution of task preparation to performance.

**Figure 2:**
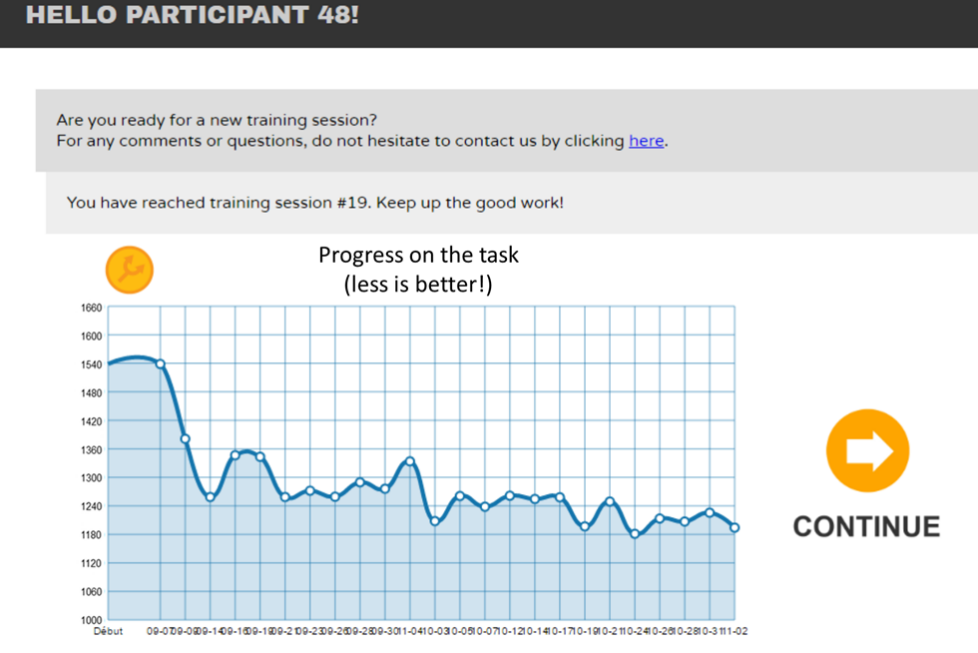
Illustration of a graph showing the progress of a fictive participant from the inhibition training group through each session. The score is the IES (Inverse Efficiency Score).

For both inhibition and updating training, visual feedback was provided for each response by changing the color of the response button to green when correct or red when incorrect. Moreover, successive correct answers were combined with positive visual feedback (ie, good, great, amazing, unbelievable) and displayed in the center of the screen below the target. At the end of each training session, an individualized graphic representation depicted the participant’s average daily performance (ie, the mean of reaction time divided by 1, minus the proportion of errors) and plot progression from the beginning of training (Figure 2).

The active control is a general knowledge quiz intervention run with E-Prime 3.0 software (Psychology Software Tools, Inc) on a laptop (Lenovo; Figure 1C). Previous studies have found that casual video games appear to be well suited as an active control condition [42,43], especially for computerized cognitive training where frequent responses are requested. Furthermore, a quiz game that does not require significant attentional control processes can appear to be a credible computerized cognitive training to the participants. An additional advantage of using a quiz game is that semantic knowledge and vocabulary are cognitive components that are unaffected by aging. As a result, semantic training was not expected to yield substantial cognitive benefits outside of the practiced task. Participants were presented with a series of 4-choice questions on 18 different topics (960 questions on food, science, geography, video games, history, sports, music, inventions, animals, movies and television series, art and literature, Canada, physics and space, monuments in the world, key historical dates, people and languages, herbs and spices, and fruit trees). The questions were adapted from OpenQuizzDB [44] or created by our research team. Each session included 2 blocks of 40 new questions. After an overall randomization of the entire pool of questions, they were displayed one by one in the same order for all participants, who had a maximum of 20 seconds to provide their response. Below each question, multiple answers (numbered 1, 2, 3, and 4) were displayed. Participants responded by pressing the corresponding number on a keypad. The selected questions were rated as medium difficulty on the OpenQuizzDB website. Positive feedback (ie, happy face emojis with the message “Bravo” or “Excellent, this is the right answer”) and a short explanation was provided following correct answers. Informative feedback (ie, “this is not the right answer”) and a short explanation was provided following incorrect answers, and displayed a second time at the end of the block.

To improve adherence, participants completed their training using individual tables, but in small groups of 6-10 individuals under the supervision of a trainer, who answered questions related to the task, helped manage technical issues, and encouraged completion of all exercises. All training sessions took place in the same room located at the CRIUGM. Five students were trained who rotated to ensure consistent supervision of training sessions.

### Baseline Characterization

At baseline, participants provided demographic information (age, sex, education), completed a Cognitive Reserve Proxy Questionnaire (CRQ) [45], Ischemic Index [46], and depression questionnaires (short version of Geriatric Depression Scale [GDS] for older adults [47] and Beck Depression Inventory II (BDI-II) for young adults). Cognition was measured with the Montreal Cognitive Assessment (MoCA) [48] and a French version of the Logical Memory Subtest from the Consortium for the Early Identification of Alzheimer’s Disease (CIMA-Q) [41] adapted from Wechsler Memory Scale [39]. Participants provided a saliva sample with the Oragene OG-500 collection kit at POST2 (V10). The sample was used to determine the single-nucleotide polymorphism rs4680 (Val^158^Met) of the *COMT* gene, the rs6265 (Val^66^Met) of the *BDNF* gene, and the rs7412 and rs429358 of the *APOE* gene.

An MRI examination was used to measure brain structure and function at the Functional Neuroimaging Unit of CRIUGM, using a Siemens Magnetom Prisma Fit 3 Tesla scanner (32-channel head coil). This will provide measures of baseline brain status, some which will be used as moderators and others as outcomes (see below for sequences used as outcomes). Sequences will be used to determine baseline intracranial volume, regional volumes (repetition time [TR]/echo time [TE] 2300/2.98 ms, Fa 9°, field of view [FOV] = 256 × 256, matrix 256 × 256, voxels 1 mm^3^, 192 slices), and volume of white matter lesions taken with the fluid attenuated inversion recovery sequence (TR/TE 9000/120 ms, Fa 90°, FOV 240 × 240, matrix 256 × 256, voxels 0.9 × 0.9 × 3 mm, 48 slices).

### Effect on the Trained Tasks

The improvements on the trained tasks will be reported using inverse efficiency scores (IESs) for each participant, which corresponds to the mean of the reaction time per session divided by the proportion of error minus one. These scores will be calculated for the 1-, 2-, and 3-back blocks separately for the updating training and separately for congruent and incongruent blocks for the inhibition training.

### Effect on Transfer Tasks

#### Transfer to Proximal Outcomes

An updating composite score and an inhibition composite score will be used as proximal and primary outcomes. The updating composite score will be computed by averaging the z-scores 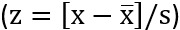 from the keep track task and the running span task, where the calculation of 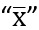 and "s" are based on the data from the PRE of each task. In the keep track task [49], participants were presented lists of 12 words from four different categories (eg, fruits, clothes, music, colors). The words were displayed one by one on a computer screen and participants were asked to report the last word belonging to each of the 4 categories. Participants updated their WM content each time they encountered a new word from the same category. The dependent variable is the proportion of words correctly recalled. In the running span task, participants were presented with lists of letters displayed one by one on a computer screen. The size of the lists varied randomly from n, where n is the participant’s letter span minus 1, to n + 6. Participants reported the n last letters in their correct order but were not informed of list’s length in advance. The dependent variable is the proportion of letters correctly recalled.

As described above for the updating composite score, the inhibition composite score will be computed by averaging z-scores 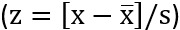 from the antisaccade task and the Victoria Stroop Test. In the antisaccade task [50], participants were asked to indicate their response with a key controlling the pointing direction of an arrow (up or down) presented in the right or left portion of a computer screen. Prior to the arrow presentation, a flashed cue will appear on the opposite side of the screen. The dependent variable is the proportion of correctly identified target arrow directions, despite the distracting cue. In the Victoria Stroop Test [51], participants were first asked to name colors using dots printed in color, noncolor words printed in color, and finally, the names of colors printed in different colors than its name. The dependent variable is the reading time for the incongruent colored words divided by the reading time for the dots printed in color. The directionality of the z-scores will be turned in the same direction as the antisaccade task (ie, higher is better).

#### Transfer to Complex WM

Performance on complex WM tasks will be measured using the alpha-span [52] and reading-span [53] tasks. In the alpha-span task, participants were asked to orally recall 5 series of words in alphabetical order rather than in the order of presentation. The words were read aloud by the assessor at a rate of 1 item per second; the size of the series corresponded to n minus 1. Prior to the alpha-span task, an individual’s n was determined as the longest sequence of words that could be repeated in the same order as presented. The dependent variable is the proportion of words recalled in the correct order. In the reading-span task, participants made yes/no semantic plausibility judgments on a series of 2-5 sentences. Following each series, participants were asked to recall orally the last word of each sentence. The dependent variables are the proportion of correct words recalled.

#### Transfer to Complex WM in Virtual Reality

An immersive virtual reality dual task was used to reflect transfer to situations that require closer to real-life cognition [10]. The task was presented with Virtools 5 (EDS Technologies) on a Dell Precision T3600 PC (Inter Xeon CPU ES-1620 0 3.60 GHz, 10-GB RAM processor, and NVIDIA GeForce GTX 600) using an HMD nVisor ST50 headset with stereoscopic vision (1280 × 1024 full color with 50° diagonal field-of-view). During this task, participants were sitting in a car as a passenger and asked to detect road signs (by pressing the left mouse button) to guide the driver to the cities “Chauminont” or “Montformeil.” Forty road signs were presented, and half were targets. At the same time, participants memorized and recalled a series of 12 words aloud, which were presented orally by the driver. The dependent variable is a dual-task score computed by averaging z-scores on the memory and detection (accuracy and reaction time) tasks.

### Brain Outcomes

#### Brain Structure

The structural sequence is a T1-weighted 3D MPRAGE sequence (TR/TE 2300/2.98 ms, Fa 9°, FOV = 256 × 256, matrix 256 × 256, voxels 1 mm^3^, 192 slices). Regional gray matter volume was measured in the prefrontal and lateral temporal cortices, basal ganglia, and hippocampi. Cortical thickness was measured in the parietal, prefrontal, and lateral temporal cortices.

#### Brain Activations

Task-related activations associated with performing updating and inhibition tasks were examined using an interleaved simultaneous multislice (accelerator factor = 6) and echo-planar imaging (TR/TE 785/30 ms, Fa 54°, FOV 192 × 192, matrix 64 × 64, voxels 3 mm^3^, 39 slices). A letter N-back task was used to assess updating activations using a block design. Three conditions were presented pseudo-randomly for a total of 15 blocks: (1) a 0-back condition serving as a control, in which a “yes” response was required upon presentation of the letter X; (2) 1-back; and (3) 2-back conditions, in which a “yes” response was required if a given letter was identical to the one presented at the *n*-back position in the sequence; and in other cases, a “no” response was expected. A 3-color Stroop task was administered to assess activity relating to inhibition. The 3 conditions, presented across 15 blocks, were as follows: (1) neutral (a string of the letter X presented in blue, green, or red font), (2) congruent (the words “BLUE,” “GREEN,” and “RED” presented in blue, green, and red font, respectively), and (3) incongruent (these same words in a colored font that does not correspond to the significance of the word). Throughout all conditions, participants also indicated the color of the font.

### Quality Control and Data Management

All assessors and supervisors received an 8-hour training session to ensure treatment adherence and harmonize data collection. They were provided with a manual that details the procedure or training. Furthermore, the first 2 participants were tested under supervision. Data were entered at the end of each wave by the assessors, who were blinded to treatment allocation. Double data entry was used for quality control for transfer cognitive tasks with manually entered scores (ie, running span, keep track, alpha span, reading span, and verbal recall in VR task). When the study was complete, the participants were contacted by phone to answer a Likert-scale questionnaire assessing their motivation associated with the intervention they received (Multimedia Appendix 1).

### Statistical Analyses

#### Sample Size

Our aim is to recruit 90 participants in total. Given an attrition rate between 10% and 16% (see [54] for a review), there would be approximately 27 participants per condition. Assuming a significance level of α=.05, a power of 0.80, and a correlation of *r*=.50 between 3 repeated measures, the G*Power 3 software for mixed designs estimates that the sample size will provide sufficient power to detect a small to medium effect (*f*=0.15). Indeed, small to medium effect sizes correspond to those observed with similar training programs in the meta-analysis by Lampit et al [55].

#### Analysis of Behavioral and Brain Outcomes

We will use a modified intention-to-treat analysis of behavioral outcomes to minimize attrition-related bias, so that all participants who have completed at least one postbaseline assessment will be analyzed. The effect of training programs on the behavioral outcomes will be analyzed with linear mixed-effects models (LMMs) as this analysis makes it possible to compare performance growth between groups and is resistant to missing values. Training improvements will be tested using IES as the dependent variable with 2 separate analyses using *session* (12 levels: session 1 to session 12) *× condition* (3 levels: IES 1-back/IES 2-back/IES 3-back) for the updating training and *session* (12 levels: session 1 to session 12) *× condition* (2 levels: IES Congruent/IES Incongruent) for inhibition training. When significant interaction effects are found, pairwise comparisons will be computed within each training group (comparing performances at sessions 6 and 12 to session 1) and between training conditions at sessions 6 and 12.

Efficacy of training to improve proximal task performances will be tested at 2 separate *time* (three levels: PRE/POST2/POST4) × *training* (3 levels: inhibition/updating/general knowledge) LMMs, using inhibition and updating composite scores as dependent variables. When significant interaction effects are found, pairwise comparisons will be computed within each training group (comparing performances at POST2 and POST4 to PRE) and between training groups (comparing performances for the inhibition and updating condition to general knowledge condition) at POST2 and POST4. To reduce the statistical power cost due to multiple testing corrections and as we have a priori hypotheses, we will also conduct LMM analyses in the absence of interaction to make a direct comparison between the performances of the general knowledge training group (control active) and the updating or inhibition training groups separately.

Efficacy of training to improve complex WM will be tested in the same way with separate time-varying LMMs (5 levels: PRE/POST1/POST2/POST3/POST4) × *training* (3 levels: inhibition/updating/general knowledge) for each task (ie, reading span, alpha span, and VR composite score).

Finally, we will use a per-protocol analysis for the neuroimaging outcomes. Structural MRI images will be analyzed with FreeSurfer 6 [56] to calculate regional cortical gray matter thickness, area, and volume, in the prefrontal, parietal and temporal cortices. Subcortical volumes were segmented for basal ganglia and hippocampi. fMRI images will be preprocessed with SPM12 [57] (realignment, slice timing, coregistration, normalization, smoothing) and will be analyzed as a block design model at subject level with a fixed-effects general linear model (GLM). The GLM will use 1 regressor for each condition task convolved with a canonical hemodynamic response function. A high-pass filter of 128 seconds will be applied to remove low frequencies. Task-related activation will be analyzed using the following fMRI contrasts: [1-back > 0-back] and [2-back > 0-back] as a measure of updating at varying task loads; and [2-back > 1-back] as a measure of load-related activation for the N-back task; [congruent > neutral] as a measure of reading-related activation; and [incongruent > neutral] and [incongruent > congruent] as measures of inhibition for the Stroop task. Whole-brain analysis will help determine task-related activation and whether alternative regions are recruited throughout or after training. Mixed ANOVAs will be carried out in regions of interest using beta weights from the activation clusters and from well-documented task-related regions. We will also adopt a parametric approach analysis, in which the first-level GLM included regressor for task block with parametric modulator for WM load.

#### Analysis of Moderators

Linear and logistic regression analyzes will evaluate the relationship between change scores (from behavioral and brain outcomes) and personal variables. As predictors, we will use: (1) education, (2) cognition at baseline; (3) scores on the CRQ; (4) motivation scale score; (5) sex; (6) genotypes (ie, BDNF, COMT, and APOE-ε4); (7) pretraining intracranial volume and white matter lesions; (8) regional gray matter volumes in the prefrontal and lateral temporal cortices; (9) basal ganglia and hippocampi; and (10) pretraining functional activations in the prefrontal and lateral temporal cortices, basal ganglia, and hippocampi. Linear multiple regression and dichotomous moderator analyzes will also be used to examine whether the CRQ’s score and the genotypes moderate the relationship between the change scores and the other predictors.

#### Analysis of Dose Effects

Dose effect will be analyzed with unconditional and conditional growth models to estimate and model the changes in behavioral and brain outcomes as training progresses.

#### Analysis of Age Differences

After ensuring that the 3 training groups are equivalent, we will first assess the effect of age prior to intervention using ANOVAs, which will compare the performance in both age groups at baseline to assess whether the intervention reduces the effect of age on behavioral and brain outcomes. Separate analyses will be used for the different outcome variables. Using additional ANOVAs, we will then compare the performance of younger participants to the POST4 performance of older participants as a function of their training group and task conditions. Performance of each training group will be compared to the younger group using pairwise comparisons. Finally, whole-brain T-tests in SPM12 will compare age groups at PRE and POST4 to observe differences in activation patterns.

### Ethics Approval and Consent to Participate

The study has been approved by the Ethics Committee for Aging-Neuroimaging Research of the Integrated University Center of Health And Social Services of South-Central-Island-of-Montreal (CIUSSS; application #CERVN17-18-02, approval May 8, 2017). Participants signed an informed consent form at their first evaluation visit.

### Security, Storage, and Confidentiality

The data are deidentified. Participants are assigned a single alpha-numeric number based on their entry in the project. All data and information are identified with this number. Brain images are processed to remove any personal information or identifier. Personal information and the key with the assigned number are kept in locked filing cabinets and available just for the principal investigator (SB). If anonymized information has to be downloaded to computers, it is kept in secured files.

### Access to Data

The data sets for this study will be made available after publication on request to the principal investigator.

### Dissemination Policy

Data will be presented in international conferences and through publications in journals with peer-reviewed committees. Study results will also be presented to the public through lay-audience talks and press releases.

## Results

The project was funded in January 2017; enrollment began in October 2017 and data collection was completed in April 2019. Data analysis has begun in June 2020 and the first results should be published by the end of 2020 or early 2021.

## Discussion

This study is a randomized double-blind controlled trial designed to examine the impact of 2 attentional control interventions, inhibition and updating, on the cognition and brain of older adults. The effect will be examined on proximal measures of inhibition and updating, complex WM measures and brain status, which will be measured with structural MRI and fMRI. Updating and inhibition are linked to age-related decline in WM [58], and have been identified as the most critical attentional control processes supporting fluid cognition [59]. Some studies have found some cognitive improvements following attentional control training in older adults (see [54] for a review). However, no study has yet to compare the relative efficacy of 2 attentional control interventions in older adults.

One strength of this study is precisely the side-by-side comparison of updating and inhibition interventions, against an active control condition. Moreover, updating and inhibition rely on distinct brain regions (eg, frontostriatal regions and right inferior frontal gyrus, respectively), which simplifies the examination of the relationship between brain and cognitive changes. This study will also establish whether inhibition and updating training programs are useful cognitive interventions to improve performance beyond the training tasks and, ultimately, to improve complex WM functioning in aging. The inclusion of a group of younger adults will help determine whether the training programs increase the performance of older adults to the level of the younger participants. Comparing younger and older adults will also clarify whether cognitive gains result from the restoration of the specialized brain regions (eg, normalization of activations relative to younger adults) or from compensative processes (eg, increased activations in specialized or alternative regions).

This study is particularly innovative due to the use of VR, which will objectively measure performance in situations that approximate real life and examine transfer in everyday situations. Transfer to real life is usually measured using self-reported questionnaires, which may lack sensitivity to intraindividual changes, especially within the short period covered by this study (eg, [60]). Finally, another strength of this study is the analyses of genetic moderators to identify and characterize responders. There has been tremendous interest recently in the potential of plasticity-related genes. A better understanding of the moderating role of genetic polymorphisms on training efficiency and transfer will help promote better adapted cognitive training programs.

The study requires many in-laboratory sessions, which may limit the recruitment of less mobile or less healthy older adults, hence reducing the generalizability of our findings. Another limitation is the absence of a no-contact control group because the cognitive gains from the active control intervention (a general knowledge quiz game) are unknown. As this training induces memory searching and potential reflexive abilities, WM could be moderately stimulated. Therefore, the differences in cognitive gains between the experimental groups and the active control group may not be as strong as expected, which would suggest a cautious interpretation of the impact of the interventions.

## Appendix

Multimedia Appendix 1 Likert scale questionnaire assessing motivation and perceptions of improvement associated with the interventions.

Multimedia Appendix 2 Previous peer-review report.

## Abbreviations

ADNI: Alzheimer’s Disease Neuroimaging Initiative
APOE-ε4: apolipoprotein ε4
BDI-II: Beck Depression Inventory II
BDNF: brain-derived neurotrophic factor
CIMA-Q: Consortium for the Early Identification of Alzheimer’s Disease
COMT: Catechol-O-methyltransferase
CRIUGM: Research Center of the Institut universitaire de gériatrie de Montréal
CRQ: Cognitive Reserve Proxy Questionnaire
fMRI: functional magnetic resonance imaging
FOV: field of view
GDS: Geriatric Depression Scale
GLM: general linear model
IES: inverse efficiency score
LMM: linear mixed-effects models
MoCA: Montreal Cognitive Assessment
SPIRIT: Standard Protocol Items: Recommendations for Interventional Trials
TE: echo time
TR: repetition time
WM: working memory
