# Supplementary material for: Assessing the Effect of Training on the Cognition and Brain of Older Adults: Protocol for a Three-Arm Randomized Double-Blind Controlled Trial (ACTOP)
Source: JMIR Res Protoc. 2020 Nov 24;9(11):e20430. doi: 10.2196/20430 (PMC7723746; doi:10.2196/20430)
Supplement: Multimedia Appendix 1 [file resprot_v9i11e20430_app1.docx]

Multimedia Appendix 1

**Questionnaire sur l’engagement et la motivation lors des entraînements**

(Précisez que ce questionnaire ne concerne que les séances d’entraînements de 30min)

**Question sur l’agréabilité des entrainements :**

- Avez-vous trouvé l’activité intéressante ? Sur une échelle de 1 (pas du tout intéressante) à 5 (très intéressante).

| 1 | 2 | 3 | 4 | 5 |
| --- | --- | --- | --- | --- |
|  |  |  |  |  |

- Trouviez-vous l’activité plaisante à réaliser ? 1 (pas du tout plaisante) à 5 (très plaisante).

| 1 | 2 | 3 | 4 | 5 |
| --- | --- | --- | --- | --- |
|  |  |  |  |  |

- Avez-vous trouvé les entraînements difficiles ? 1 (très difficile) à 5 (très facile).

| 1 | 2 | 3 | 4 | 5 |
| --- | --- | --- | --- | --- |
|  |  |  |  |  |

**Question sur l’efficacité perçue des entrainements :**

- Aviez-vous l’impression de travailler fort pour vous améliorer ? 1 (jamais) à 5 (en permanence).

| 1 | 2 | 3 | 4 | 5 |
| --- | --- | --- | --- | --- |
|  |  |  |  |  |

- Aviez-vous eu l’impression de vous être amélioré(e) lors des entraînements ? 1 (pas du tout) à 5 (énormément).

| 1 | 2 | 3 | 4 | 5 |
| --- | --- | --- | --- | --- |
|  |  |  |  |  |

- Trouviez-vous l’activité pertinente pour tenter d’améliorer la santé cognitive ? 1 (pas du tout pertinente) à 5 (très pertinente).

| 1 | 2 | 3 | 4 | 5 |
| --- | --- | --- | --- | --- |
|  |  |  |  |  |
