# Supplementary material for: Assessing the Effect of Training on the Cognition and Brain of Older Adults: Protocol for a Three-Arm Randomized Double-Blind Controlled Trial (ACTOP)
Source: JMIR Res Protoc. 2020 Nov 24;9(11):e20430. doi: 10.2196/20430 (PMC7723746; doi:10.2196/20430)
Supplement: Multimedia Appendix 2 [file resprot_v9i11e20430_app2.pdf]

Conseil de recherches en sciences naturelles et en génie du Canada
Natural Sciences and Engineering Research Council of Canada

350, rue Albert
Ottawa, Canada
K1A 1H5

350 Albert Street
Ottawa, Canada
K1A 1H5

PROTÉGÉ

Le 27 janvier 2017

Sylvie Belleville
Université de Montréal
Arts et des sciences, faculté des

Identificateur personnel commun (IPC) : 1016095

Numéro de la demande : RGPIN-2016-06132

Programme : Programme de subventions à la découverte

Groupe d'évaluation : Systèmes et fonctions biologiques - 1502

Titre : Mémoire et contrôle cognitif pour un vieillissement optimal : études sur l'effet des interventions cognitives, les mécanismes cérébraux mis en jeu et les processus favorisant le transfert

Nous avons le plaisir de vous annoncer que le Conseil de recherches en sciences naturelles et en génie du Canada (CRSNG) a révisé le montant de votre subvention à la découverte compte tenu des nouveaux fonds attribués dans le budget fédéral de 2016.

Le sommaire de la subvention et les conditions qui s'appliquent à cette subvention sont décrites dans les *Modalités et conditions de la subvention* ci-jointes. Veuillez les lire attentivement. Vous y trouverez des renseignements importants sur votre subvention.

Le Programme de subventions à la découverte du CRSNG s'appuie sur la détermination de la communauté scientifique à veiller à ce que le CRSNG prenne les décisions appropriées concernant l'octroi des subventions, par suite des recommandations formulées par les pairs évaluateurs. Le CRSNG s'attend à ce chaque titulaire d'une subvention participe au processus d'évaluation par les pairs, à titre d'évaluateur, si on le lui demande. De plus, l'établissement du titulaire devrait reconnaître la participation de ce dernier comme une importante contribution aux activités de recherche au Canada et une preuve de l'excellence du chercheur.

Cette notification de décision annule et remplace celle déjà émise.

Pierre J. Charest, Ph.D.
Vice-président
Subventions de recherche et bourses

c. c. : Responsable des subventions de recherche, Université de Montréal
Responsable des finances, Université de Montréal

Canada

SOMMAIRE DE LA SUBVENTION

**Candidat :**	Sylvie Belleville
**Identificateur personnel commun (IPC) :**	1016095
**Numéro de la demande :**	RGPIN-2016-06132
**Titre de la subvention** :	Mémoire et contrôle cognitif pour un vieillissement optimal : études sur l'effet des interventions cognitives, les mécanismes cérébraux mis en jeu et les processus favorisant le transfert
**Organisation administratrice :**	Université de Montréal
**Montant de la subvention :**	(1/5) 2016/2017 $51,000<br>(2/5) 2017/2018 $51,000<br>(3/5) 2018/2019 $51,000<br>(4/5) 2019/2020 $51,000<br>(5/5) 2020/2021 $51,000

MODALITÉS ET CONDITIONS DE LA SUBVENTION

Le CRSNG a le plaisir de vous offrir une subvention à l'appui des activités énoncées dans votre demande pour le montant et la durée décrits ci-dessus.

Lorsque vous vous prévalez des fonds accordés, vous confirmez que pendant la période de validité de la subvention/bourse, vous acceptez de respecter les modalités et conditions suivantes.

1. Vous utilisez la subvention uniquement aux fins pour lesquelles elle a été accordée.
2. Vous fournissez des renseignements justes, complets et exacts dans tous les documents liés à votre subvention et vous vous présentez et présentez vos travaux et vos réalisations conformément aux normes du domaine visé par la demande.
3. Vous vous assurerez que les personnes qui sont nommées dans tous les documents liés à votre subvention ont accepté que leur nom y soit inclus et que leurs renseignements personnels y soient divulgués.
4. Si vous êtes un cocandidat, vous acceptez que le candidat administre la subvention ou la bourse au nom du groupe ou de l'équipe.
5. Vous n'avez pas été déclaré, à l'heure actuelle, non admissible à demander ou à détenir des fonds du CRSNG, du Conseil de recherches en sciences humaines du Canada (CRSH) ou des Instituts de recherche en santé du Canada (IRSC), ou de tout autre organisme voué à la recherche ou organisme de financement de la recherche, au pays ou à l'étranger, pour des motifs de non-respect des politiques sur la conduite responsable de la recherche comme des politiques en matière d'éthique, d'intégrité ou de gestion financière. Si, en tout temps, vous êtes déclaré non admissible pour l'une ou l'autre des raisons susmentionnées, vous devez aviser immédiatement par écrit un représentant officiel de votre établissement et le CRSNG.

6. Vous respectez le Cadre de référence des trois organismes sur la conduite responsable de la recherche, qui comprend, sans s'y limiter, le Guide d'administration financière des trois organismes, le *Guide des programmes destiné aux professeurs* et la Politique inter-conseils sur l'intégrité dans la recherche ainsi que toute modification que le CRSNG pourrait adopter.
7. Si vous êtes ou avez été (à n'importe quel moment au cours des 12 derniers mois) un fonctionnaire fédéral, vous avez respecté et continuerez de respecter le Code de valeurs et d'éthique du secteur public.
8. Vous consentez au partage de l'ensemble ou d'une partie des renseignements concernant la demande et la subvention ou la bourse, y compris des renseignements personnels, avec les trois organismes subventionnaires fédéraux (le CRSNG, le CRSH et les IRSC) et tout établissement postsecondaire auquel vous êtes affilié ou pourriez le devenir.
9. Vous informerez immédiatement par écrit le CRSNG et les responsables officiels de votre établissement de tout changement à l'égard de votre statut d'admissibilité.
10. Vous informerez les représentants de votre établissement de tout changement dans la nature de la recherche qui pourrait avoir une incidence sur la certification ou sur les approbations précisées à la section 2.4 Exigences des organismes concernant certains types de recherche, du Cadre de référence des trois organismes sur la conduite responsable de la recherche.
11. Vous informez le CRSNG et les représentants de votre établissement de tout changement dans la nature de la recherche qui peut exiger une évaluation environnementale ou l'utilisation de cellules souches pluripotentes humaines. Vous n'entreprenez pas ces activités de recherche exigeant une certification supplémentaire avant d'avoir obtenu l'approbation du CRSNG.
12. Vous informez les représentants de votre établissement si vos plans de recherche changent pour inclure de nouvelles activités utilisant des sujets humains ou exigeant l'utilisation d'animaux. Vous n'entreprenez pas ces activités de recherche avant d'avoir obtenu l'approbation de l'établissement.
13. Si vous décidez de commercialiser des résultats de la recherche, vous divulguez à votre établissement toute propriété intellectuelle (PI) potentielle découlant de la recherche.
14. Si vous divulguez toute PI potentielle découlant de la recherche, vous et votre établissement faites en sorte que le Canada retire le plus grand avantage économique possible de l'activité commerciale qui en résultera. Cette exigence relative à la divulgation ne vise pas à remplacer la politique en matière de droits de propriété intellectuelle déjà en vigueur au sein de l'établissement d'enseignement.

En outre, vous comprenez ce qui suit.

15. Le CRSNG se réserve le droit de reporter ou d'annuler le versement d'une subvention si le besoin continu de fonds n'est pas dûment justifié.
16. La subvention est accordée sous la réserve de la disponibilité des fonds. Le CRSNG peut réduire ou annuler la subvention.
17. La Loi sur l'accès à l'information et la Loi sur la protection des renseignements personnels (AIPRP) et l'utilisation et la divulgation des renseignements personnels fournis au CRSNG ont trait aux renseignements que le CRSNG vous demande de lui fournir et qu'il recueille à votre sujet.

Modalités et conditions supplémentaires :

18. Vous respectez toute autre condition relative aux dépenses énoncée dans le message au candidat.

Consentement à la divulgation de renseignements personnels : Vous comprenez que le maintien de la confiance du public à l'égard de l'intégrité des chercheurs est essentiel à l'établissement d'une société axée sur le savoir. Lorsque vous acceptez le financement des IRSC, du CRSNG ou du CRSH, vous confirmez avoir lu toutes les politiques de ces organismes qui se rapportent à vos travaux de recherche et

que vous acceptez de les respecter, y compris le Cadre de référence des trois organismes sur la conduite responsable de la recherche. Dans le cas de violation grave des politiques des organismes subventionnaires, ces derniers peuvent divulguer publiquement tout renseignement pertinent qui est d'intérêt public, y compris votre nom, la nature de la violation, le nom de l'établissement où vous avez travaillé au moment de la violation, le nom de l'établissement où vous travaillez actuellement et les recours pris à votre endroit. Vous acceptez qu'il s'agisse d'une condition pour présenter une demande aux organismes ou pour recevoir des fonds des organismes et vous consentez à cette divulgation. Si vous refusez que vos renseignements personnels soient divulgués, vous ne pourrez pas participer à cette subvention ou à cette bourse.

Remarque : Si vous croyez ne pas pouvoir respecter les modalités susmentionnées, communiquez immédiatement avec les responsables officiels de votre établissement ou le personnel du CRSNG responsable du programme. N'acceptez pas les modalités et n'utilisez pas les fonds qui vous ont été versés ou qui ont été versés à l'établissement d'accueil avant que vous soyez certain que vous pouvez respecter toutes les exigences.

External Reviewer Report - Application for a Grant
Rapport de l'évaluateur externe - Demande de subvention

**Family name, given name and initials(s) of applicant / Nom de famille, prénom et initiale(s) du candidat:**<br><br>Belleville, Sylvie	**Application no. / N° de la demande:**<br><br>RGPIN-2016-06132
**Type of grant / Type de subvention:** RGPIN	**Committee / Comité:**<br>1502<br>Biological Systems and Functions / Systèmes et fonctions biologiques

Confidence Level / Niveau de confiance

I rate my ability to assess this particular application as / Mes compétences pour évaluer cette demande sont:

High / Elevées: X Satisfactory / Satisfaisantes: Low / Faibles:

EVALUATE EACH ITEM BELOW AND EXPLAIN YOUR ASSESSMENT BY REFERRING TO THE INFORMATION PROVIDED IN THE APPLICATION THAT LEADS YOU TO YOUR CONCLUSIONS. / ÉVALUEZ CHACUN DES POINTS CI-DESSOUS ET EXPLIQUEZ VOTRE ÉVALUATION EN CITANT LES RENSEIGNEMENTS PRÉSENTÉS DANS LA DEMANDE QUI VOUS ONT MENÉ À VOS CONCLUSIONS.

Excellence of the Researcher: Applicants must present evidence of meaningful research contributions to the natural sciences and engineering field **in the past six years**. Account for any legitimate delays in research.

Excellence du chercheur : Le candidat doit prouver qu'il a apporté des contributions appréciables en sciences naturelles ou en génie au cours **des six dernières années**. Tenez compte des retards légitimes dans la recherche.

Describe the applicant's strengths and weaknesses related to / Décrivez les points forts et les points faibles du candidat en ce qui concerne :

the knowledge, expertise and experience of the researcher in the natural sciences and engineering, and their stature in the field; / les connaissances, l'expertise et l'expérience du chercheur en sciences naturelles ou en génie et sa réputation dans le domaine;

Dr Belleville has a strong track record of publications in brain aging both normal and pathological which has been recognised as significant for the field of cognitive aging. Even though most of her funding is for pathological aging, I counted 21 articles dealing with healthy brain aging including a number of methodological studies to develop neuropsychological instruments. Among the recent publications I note

Sylvain-Roy and Belleville (2015) and Sylvain-Roy et al. (2015) that examine attentional control in normal elderly as well as Byer, deBoysson & Belleville 2014 that examined how to improve multitasking in older people. I also thought that the Belleville et al (2014) paper that examined functional brain imaging changes correlated with brain training modalities in healthy aging was particularly interesting.

the quality and impact of their research in the natural sciences and engineering. / la qualité et l'incidence de ses recherches en sciences naturelles ou en génie.

Dr Belleville has published a large number of articles in her carreer, she has also contributed significantly to the field. Her work on normal aging and normal cognitive processes appears to be well received.

Merit of the Proposal: The proposal must clearly present a **program** of research in the natural sciences and engineering. The program must not be limited to the development of specific applications of existing knowledge; it must promise an original and innovative contribution.

Mérite de la proposition : La proposition doit clairement présenter **un programme** de recherche en sciences naturelles ou en génie. Le programme ne doit pas être limité à la mise au point d'applications particulières de connaissances existantes; il doit mener à une contribution originale et innovatrice.

Describe the strengths and weaknesses of the proposal related to: / Décrivez les points forts et les points faibles de la demande en ce qui concerne :

the originality, innovation of the proposal, and the extent to which it suggests and explores novel concepts and lines of inquiry; / l'originalité et le caractère novateur de la proposition et la mesure dans laquelle la recherche est susceptible d'aborder des concepts et des pistes de recherche nouveaux;

The research program is well developed and uses current concepts in brain aging as well as a wide range of experimental procedures. It is clear and well written and provide enough details to predict that it will be successfully completed. I have a few concerns presented for each experiment – some of which are related to current limitations of the field itself.
The first experiment raise an interesting question regarding the impact of training on brain functions in older people in the context of the larger question of brain plasticity during aging. The experiment's strength is trying to increase the efficiency of core cognitive processes such as attention (and its mirror function inhibition). One question I had is whether the individual improvement in performance at the proposed tasks would be taken as the efficiency of the training and used as a covariate. One criticism I have (but it applies to the whole field) is that very specific tasks (and task modalities) are used for training. I would assume that the limited variability in training limits its effect on the brain. The physical exercise analogy would be that doing hundreds of bicep curls will not improve the functionality of other muscle groups. Following this analogy, a protocol that uses multiple and diverse muscular exercises has been proven to be more beneficial for improvement in physical abilities in aging. Beyond this issue, the experiment is interesting and the methodology up to date.

The second experiment proposes to examine changes in functional imaging observations under a number of tasks before and after cognitive training to determine the functional changes associated with training. I found the introduction to that experiment a bit old-fashioned in that it appears to propose that mostly increases in functional activity will be taken as demonstrating a beneficial outcome of cognitive training. Today, most

researchers would mention both increases and decreases in activations as a possible positive outcome. Many experiments have now shown that better cognitive performance can be associated with decreased activation because the brain becomes more efficient. On the other hand increased activation might signal more difficulty to perform tasks. The data analysis section is too short to determine if an appropriate analysis is planned. Of course, the 5-page format of the proposal constrains the amount of details on that topic and a lengthier description may very well dispel my doubts.

Experiment 3 proposes to examine if the impact of training on specific tasks is greater when it recruits the same brain areas as the tests in the evaluation (post) phase. I was left with the impression that this was already part of experiment 2. I guess one could see experiment 3 as specifying one additional hypothesis using the same data set.

The fourth experiment is tantalizingly the most interesting as it raise the most important question: does brain training using specific tasks as presented in this proposal really make a difference in the real world? Unfortunately, this section was underdeveloped compared to the previous experiments. However, I thought that the development of a virtual office environment was innovative as a testing tool.

This is an experienced researcher with demonstrated expertise in that area so I don't expect major problems for feasibility.

the anticipated significance of and expected contributions to research, and potential for impact; / l'importance prévue des travaux et les contributions prévues à la recherche, et les possibilités de répercussions;

There is a good probability of potential impact in the field of cognitive aging

the clarity and scope of the short and long-term objectives, methodology and feasibility. / la clarté et la portée des objectifs à court et à long terme du programme de recherche, la méthodologie et à la faisabilité.

The clarity and scope are adequate

Contribution to the Training of Highly Qualified Personnel (HQP): Contributions **in the past six years** to quality training at all levels (undergraduate students to postdoctoral fellows as well as technicians and research associates) are valued. Assessment of contributions to the training of HQP includes both the applicant's track record and the plans for future training.

Contribution à la formation de personnel hautement qualifié (PHQ) : On accorde de l'importance aux contributions à la formation de qualité à tous les niveaux (les étudiants de premier cycle et des cycles supérieurs, les stagiaires postdoctoraux, les techniciens et les attachés de recherche) au cours **des six dernières années.** L'évaluation des contributions à la formation de PHQ doit tenir compte des contributions antérieures et de la formation prévue.

Describe the applicant's strengths and weaknesses related to: / Décrivez les points forts et les points faibles du candidat en ce qui concerne :

the quality and impact of contributions to the training of HQP during the last six years; / la qualité et

**l'incidence des contributions à la formation de PHQ au cours des six dernières années;**<br><br>The applicant has a strong record of HQP training.
**the appropriateness of the proposal for the training of HQP in the natural sciences and engineering, and a clear description of the training plan. / la pertinence de la proposition au regard de la formation de PHQ en sciences naturelles ou en génie et la description claire du plan de formation.**<br><br>The plan is appropriate. I note that within this large research group, students meet one-to-one with the PI at least once a month.
**Additional comments on the application: / Commentaires supplémentaires sur la demande :**

Natural Sciences and Engineering Research Council of Canada
Conseil de recherches en sciences naturelles et en génie du Canada

350 Albert Street
Ottawa, Canada
K1A 1H5

350, rue Albert
Ottawa, Canada
K1A 1H5

MESSAGE TO APPLICANT	MESSAGE AU CANDIDAT
This message represents the consensus opinion of the evaluation group that reviewed your application.	Ce message reflète le consensus de l'opinion du groupe d'évaluation qui a évalué votre demande.

Applicant's Name, Appl. ID, Institution / Nom de famille, numéro de la demande, établissement du candidat	Belleville, Sylvie, RGPIN-2016-06132, Université de Montréal
Type of Grant / Genre de subvention	Discovery Grants Program - Individual<br>Programme de subventions à la découverte - individuelles
Evaluation Group / Groupe d'évaluation	Biological Systems and Functions<br>Systèmes et fonctions biologiques
Application Title / Titre de la demande	Mémoire et contrôle cognitif pour un vieillissement optimal : études sur l'effet des interventions cognitives, les mécanismes cérébraux mis en jeu et les processus favorisant le transfert

The Evaluation Group rated your application as follows / Le groupe d'évaluation a attribué les cotes suivantes à votre demande de subvention :

Excellence of the Researcher / Excellence du chercheur :	Remarquable

Le Groupe d'évaluation a évalué les contributions du candidat aux domaines des sciences naturelles et du génie en se basant sur plusieurs facteurs, notamment les connaissances du candidat, son expertise, son expérience et sa réputation dans le domaine, ainsi que la qualité et l'incidence de ses réalisations en recherche au cours des six dernières années.

Merit of the Proposal / Mérite de la proposition :	Très Élevé

Le Groupe d'évaluation a évalué un certain nombre de facteurs tels que (i) l'originalité et le caractère novateur de la recherche proposée; (ii) l'importance des travaux et contributions prévues à la recherche; (iii) la clarté et la portée des objectifs; (iv) la clarté et la pertinence de la méthode; (v) la faisabilité du plan de recherche; (vi) ainsi que la pertinence et la justification du budget.

Training of Highly Qualified Personnel / Formation de personnel hautement qualifié :	Très Élevé

Le Groupe d'évaluation a évalué la qualité et l'incidence de la formation de personnel hautement qualifié (PHQ) au cours des six dernières années, ainsi que la pertinence du programme de recherche pour la formation de PHQ et les plans proposés pour la formation future.

Cost of Research / Coût de la recherche :	Normal

Additional Comments / Commentaires additionnels :

Dans le concours de 2016, le groupe d'évaluation a évalué en tout 467 demandes de subvention à la découverte. Ces demandes ont été évaluées selon les directives qui figurent dans le Manuel d'évaluation par les pairs du CRSNG, affiché dans le site Web du CRSNG.

Pour chaque demande, le CRSNG communique avec des évaluateurs externes afin de les inviter à réaliser une évaluation et à présenter un rapport. Les évaluateurs externes sont généralement des personnes suggérées par le candidat, ainsi que d'autres personnes choisies par les membres du Groupe d'évaluation. Le choix de participer à une évaluation et de présenter un rapport appartient aux évaluateurs externes.

Le groupe d'évaluation examine, à la lumière des critères de sélection, les renseignements que renferment la demande de subvention et le CVC. Les membres du Groupe d'évaluation réalisent une évaluation indépendante de chacune des demandes qui leur ont été assignées avant les réunions d'évaluation. Au cours de la réunion du Groupe d'évaluation, chaque demande fait l'objet d'une discussion approfondie. Les rapports des évaluateurs externes ne sont qu'un des éléments dont les membres du Groupe d'évaluation tiennent compte dans leur évaluation; ceux-ci se fondent avant tout sur leur propre évaluation pour formuler leurs recommandations.

Dans certains cas, il pourrait sembler au candidat que les rapports des évaluateurs externes ne concordent pas avec les commentaires et les recommandations formulés par le Groupe d'évaluation. En effet, il faut garder à l'esprit que les rapports des évaluateurs externes ne sont qu'un des éléments dont les membres du groupe d'évaluation tiennent compte dans leur propre évaluation. Par ailleurs, les évaluateurs externes lisent au plus trois propositions reçues par le CRSNG au cours d'une année donnée; dans la plupart des cas, ils n'en lisent qu'une seule. Par conséquent, les évaluateurs externes n'ont pas la même vision d'ensemble que les membres du groupe d'évaluation, qui ont examiné en moyenne 50 demandes de subvention à la découverte dans le cadre du concours de cette année.
